# Interaction of nanoplastics with platelets: activation and fibrin clot formation

**DOI:** 10.3389/fphar.2026.1897581

**Published:** 2026-08-24

**Authors:** A. Trostchansky, M. Alarcón

**Affiliations:** 1 Departamento de Bioquímica and Centro de Investigaciones Biomédicas (CEINBIO), Facultad de Medicina, Universidad de la República, Montevideo, Uruguay; 2 Thrombosis Research Center and Healthy Aging, Universidad de Talca, Talca, Chile; 3 Department of Clinical Biochemistry and Immunohematology, Faculty of Health Sciences, Universidad de Talca, Talca, Chile

**Keywords:** clot, microplasitcs, nanoplastic (NP), platelets, thrombosis

## Abstract

**Introduction:**

Environmental exposure to micro- and nanoplastics (NPs) is an emerging toxicological concern, with recent evidence demonstrating their presence in human blood and vascular tissues. Although plastic‐associated chemicals are known to induce oxidative stress and mitochondrial dysfunction, the direct impact of circulating NPs on blood cell function and redox‐sensitive vascular processes remains poorly defined. Therefore, this study investigated the interaction between 100 nm carboxylated polystyrene nanoplastics (PS‐NPs) and human platelets, which are key regulators of vascular homeostasis and thromboinflammation.

**Methods:**

The interaction and uptake of PS‐NPs by human platelets were assessed using flow cytometry and fluorescence microscopy. To evaluate platelet activation, we measured cytoskeletal remodeling (via side scatter) and dense granule secretion (via CD63 externalization). Additionally, whole blood assays were conducted under calcium‐permissive conditions to determine the impact of PS‐NPs on the formation and structure of fibrin networks.

**Results:**

PS‐NPs rapidly associated with human platelets in a time‐ and concentration‐dependent manner, reaching near‐maximal uptake within 10 minutes. Exposure to PS‐NPs induced marked platelet activation, evidenced by significant cytoskeletal remodeling and concentration‐dependent CD63 externalization at levels comparable to those induced by thrombin stimulation. Furthermore, in whole blood, PS‐NPs promoted the formation of dense fibrin networks and were actively incorporated into the resulting fibrin matrix.

**Discussion:**

The rapid nature of PS‐NP–platelet interactions suggests high‐capacity physicochemical adsorption, potentially mediated by membrane lipid microdomains and protein corona formation. Notably, the observed procoagulant phenotype is consistent with the perturbation of redox‐sensitive platelet activation pathways. Collectively, these findings identify platelets as direct cellular targets of nanoplastics and demonstrate that circulating particulate matter can acutely promote prothrombotic responses. These data support a model in which nanoplastic exposure contributes to cardiovascular risk through the dysregulation of redox‐dependent vascular signaling.

## Introduction

Since the mid-twentieth century, global plastic production has increased exponentially, resulting not only in a growing environmental burden of plastic waste but also in the generation of microscopic plastic particles known as microplastics (MPs). These are typically defined as particles smaller than 5 mm, while nanoplastics (NPs) are even smaller, measuring less than 1 µm. Plastic fragments have become ubiquitous across marine, terrestrial, and atmospheric environments, originating either as primary MPs—such as microbeads used in cosmetics and industrial precursors—or as secondary MPs, which form through degradation processes like ultraviolet radiation, mechanical abrasion, and incomplete biodegradation of larger plastic debris (Leslie et al., 2022; Ragusa et al., 2021; Kannan and Vimalkumar, 2021).

Humans are exposed to plastic fragments via multiple routes, including ingestion through contaminated food and water, inhalation of airborne particles and fibers, and dermal contact. Biomonitoring studies have confirmed the presence of plastic particles across various human biological matrices, including blood, placenta, and other tissues (Leslie et al., 2022; Ragusa et al., 2021). Given their pervasive nature, structural longevity, and potential to interact with biomolecules, these particles raise significant toxicological concerns (Aliya et al., 2025).

The toxic effects of plastic fragments are determined by both their physical characteristics and their surface chemistry. The small size and irregular morphology of micro- and nanoplastics facilitate tissue penetration and cellular accumulation, potentially leading to inflammatory responses and localized cytotoxicity (Rolli, 2023; Shiwakot et al., 2022; Zheng et al., 2024). Furthermore, exposure to these particles can induce systemic oxidative stress, mitochondrial dysfunction, and the disruption of vascular endothelial homeostasis (Rolli, 2023). These interactions are linked to broader cellular damage and organ-system toxicities, primarily mediated through the generation of reactive oxygen species (ROS) (Zheng et al., 2024).

Recent studies have highlighted the potential for circulating plastic fragments to be phagocytosed by immune cells in the bloodstream, causing physical obstructions in cerebral capillaries, restricting local blood flow, and inducing neurobehavioral abnormalities in murine models (Huang et al., 2025). This finding underscores the potential for plastic fragments to disrupt vascular physiology via both mechanical and cellular mechanisms. However, the specific pathways through which circulating plastic particles interact with blood components, such as platelets, to initiate acute prothrombotic events remain poorly understood.

Particularly concerning is the impact of micro- and nanoplastics on platelet function and the coagulation cascade. Studies have shown that these particulate contaminants can directly enhance platelet activation, engagement, and aggregation, contributing to a prothrombotic state that elevates the overall risk of cardiovascular diseases (CVDs) (Zheng et al., 2024; Lee, 2024; Maitz et al., 2025). Because platelets play a central role in hemostasis, inflammation, and vascular pathology, micro- and nanoplastics have emerged as critical, newly recognized contributors to cardiovascular risk (Maitz et al., 2025; Smyth et al., 2015).

Understanding the complex mechanisms underlying human exposure, bioavailability, and cellular interactions with plastic fragments and their chemical additives is critical for assessing their impact on cardiovascular health and overall systemic well-being. Emerging evidence underscores the urgent need for further investigation into the health risks posed by environmental and human exposure to plastic particles, AA, BPA, and similar compounds, as well as for regulatory measures to mitigate these risks.

## Materials and methods

Polystyrene nanoparticles (fluorescent 505/515 nm, carboxyl-modified; Thermo Fisher Scientific, United States) were used for all experiments. Sterile phosphate-buffered saline (PBS, 1×) was used for nanoparticle dilution, and particle dispersion was enhanced using a Clifton Ultrasonic Bath (Clifton, NJ, United States). Tyrode’s buffer without calcium, acid-citrate-dextrose (ACD), and 3.2% sodium citrate were used for platelet and whole blood handling. Human platelet identification and activation markers included anti-human CD41-APC-Cy7-A and CD63-PE antibodies (BD Biosciences, San Jose, CA, United States). Flow cytometric analyses were performed using a BD FACS Lyric cytometer, and microscopy was conducted using an Axio Examiner Z1 fluorescence microscope, equipped with an Axiocam 506 camera. Image processing and quantitative analyses were performed using ImageJ software (NIH; version 1.26t). All centrifugations were conducted using an Eppendorf 5804 centrifuge (Hamburg, Germany). Platelet counts were obtained using a Mindray BC-3000 Plus hematology analyzer. Calcium chloride (CaCl_2_, 100 mM) was used to induce coagulation in whole-blood assays.

### Nanoparticle preparation

Polystyrene nanoparticles (PS-NPs) of 100 nm diameter (fluorescent beads, 505/515 nm), with an anionic surface chemistry (carboxyl-modified, catalog: F8803, Thermo Fisher Scientific, United States), were used in this study as representative particles of plastic fragment contaminants. PS-NPs were diluted in sterile-filtered phosphate-buffered saline (PBS, 1X). To reduce particle agglomeration, the PS-NPs were sonicated for 1 min using a Clifton Ultrasonic Bath (Clifton, NJ), followed by vortexing for 1 min before dilution and just before use (Smyth et al., 2015).

### Platelet isolation and preparation

Platelet samples were prepared according to a standardized protocol established by our laboratory (Burgos et al., 2025), with minor modifications. Venous blood (10 mL) was collected from healthy volunteers into syringes containing acid-citrate-dextrose (ACD; 4:1 v/v). The blood was first centrifuged at 250 *g* for 12 min (without brake or accelerator; Eppendorf 5804, Hamburg, Germany) to isolate the platelet-rich plasma (PRP) fraction. The PRP was then centrifuged at 900 *g* for 8 min. The plasma supernatant was removed, and the platelet pellet was resuspended in Tyrode’s buffer without calcium, supplemented with ACD at a 5:1 v/v ratio. Platelet count was determined using a hematology counter (Mindray BC-3000 Plus, Japan). All donors were medication-free for at least 10 days before blood collection and exhibited no signs of bleeding disorders or acute illness. Samples exhibiting hemolysis or lipemia were excluded from analysis. Written informed consent was obtained from all participants before enrollment. The study protocol was approved by the Ethics Committee of the Faculty of Health Sciences, Universidad de Talca, and complied with the principles of the Declaration of Helsinki (18th World Medical Assembly, Helsinki, 1964).

### Flow cytometric analysis

Flow cytometric analysis was performed using a BD FACS Lyric flow cytometer (BD Biosciences). Data collection was conducted until 10,000 total events were recorded. Forward scatter (FSC), side scatter (SSC), and fluorescence data were obtained using a logarithmic scale configuration. All buffers were double-filtered through a 0.22 μm filter. Representative data are presented in all figures. The platelet population (>99%) in washed platelets was identified using anti-human CD41-APC-Cy7-A antibody (BD Biosciences, San Jose, CA, United States). Data were expressed as mean fluorescence intensity (MFI) (Recabarren-et al., 2018).

Washed platelets (1 mL) were treated with varying concentrations of PS-NPs (0–200 μg) or vehicle control for 10 min. Platelet activation was quantified using an antibody against CD63-PE, a marker of platelet degranulation. The potential interference of PS-NPs with the assay was assessed by measuring light scattering in the absence of cells (Supplementary Figure S1).

### Fluorescence microscopy

For bead binding and localization studies, fluorescence microscopy images were captured using the ×40 objective lens of an Axio Examiner Z1 fluorescence microscope (Zeiss). The images were subsequently analyzed using ImageJ software (version 1.26t, NIH). Image acquisition was carried out using an Axiocam 506 digital camera (Jena, Germany), and images were processed with ZEN2.3 Pro software (Jena, Germany).

### Whole blood coagulation assay

For the whole blood coagulation assays, blood was collected from healthy human volunteers using 3.2% sodium citrate as an anticoagulant to preserve cellular and plasma components. Briefly, citrated whole blood was aliquoted into 500 μL samples, treated with 10 μg of PS-NPs or vehicle, and coagulation was induced by adding calcium chloride (100 mM CaCl_2_) to achieve final concentrations of 2 mM, 4 mM, and 12 mM, with the 12 mM sample serving as the positive control. All samples were incubated at 37 °C for 30 min. Clotting was visually assessed by gently inverting the tubes. Clots formed under the 4 mM Ca^2+^ + PS-NPs condition were subsequently analyzed for physical properties: clots were carefully isolated, blotted dry, and weighed using a precision balance. Arbitrary clot size (AU) was measured using ImageJ software.

### Statistical analysis

Platelet assays were performed using blood samples from six independent healthy volunteers, a sample size deemed appropriate based on the high reproducibility and low inter-individual variability of *ex vivo* platelet assays (Burgos et al., 2025). Each volunteer served as their own control, with platelet responses measured before and after exposure to PS-NPs, allowing for paired analysis to minimize variability and enhance statistical power. Data were analyzed using GraphPad Software version 6.0 (La Jolla, CA, United States). Two or more measurements were performed for each test, and data were expressed as mean ± standard error of the mean (SEM). Differences between groups were analyzed using one-way ANOVA, with p values <0.05 considered statistically significant. The data were confirmed by a low inter-individual coefficient of variation (CV = 10.0%) and an intra-individual technical CV of 4.3%, indicating that the observed biological effect is highly reproducible and less prone to confounding inter-individual fluctuations.

## Results

### Rapid association of polystyrene nanoplastics with human platelets

Washed human platelets were identified by flow cytometry using forward scatter (FSC), side scatter (SSC), and platelet-specific fluorescence, allowing exclusion of debris and non-platelet events (Supplementary Figure S1). Platelets were exposed to fluorescent PS-NPs, and platelet-associated fluorescence was quantified over time.

**FIGURE 1:**
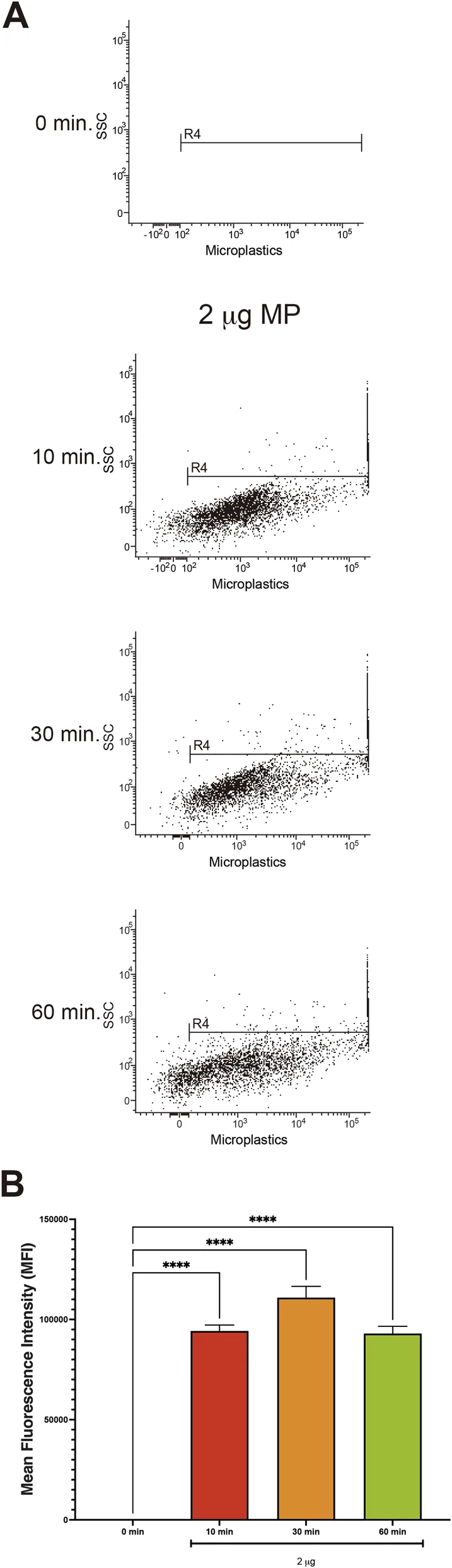
Kinetics of polystyrene nanoplastic (PS-NPs) association with washed human platelets. **(A)** Flow cytometry analysis of washed human platelets exposed to 2 μg of fluorescently labeled PS-NPs over time (0, 10, 30, and 60 min). Dot plots show the relationship between Side Scatter (SSC, Y-axis) and PS-NPs fluorescence intensity (X-axis, logarithmic scale). The 0-min time point serves as an unexposed control, defining baseline fluorescence. Exposure to PS-NPs leads to a time-dependent increase in both fluorescence (PS-NPs uptake) and SSC (reflecting enhanced internal complexity), indicating PS-NP association. Region R4 identifies platelets positive for PS-NPs fluorescence. **(B)** Mean Fluorescence Intensity (MFI) of washed platelets following PS-NPs exposure at 0, 10, 30, and 60 min. Data are presented as mean ± SEM; ****p < 0.0001vs. 0 min by one-way ANOVA with Dunnett’s test.

Exposure to 2 μg PS-NPs induced a rapid increase in platelet-associated fluorescence, accompanied by a parallel rise in SSC (Figure 1A). The proportion of PS-NPs–positive platelets increased sharply during the initial minutes of exposure and reached a plateau by approximately 10 min (Figure 1B). Mean fluorescence intensity (MFI) was significantly increased at all post-exposure time points compared with baseline (p < 0.0001), with no further increase beyond 10 min.

At a supraphysiological concentration (200 μg), maximal platelet-associated fluorescence was achieved within 10 min, with no additional increase upon prolonged incubation (Supplementary Figure S2). Based on these kinetics, a 10-min exposure was used for all subsequent experiments.

### PS-NPs induce platelet activation and cytoskeletal remodeling

**FIGURE 2:**
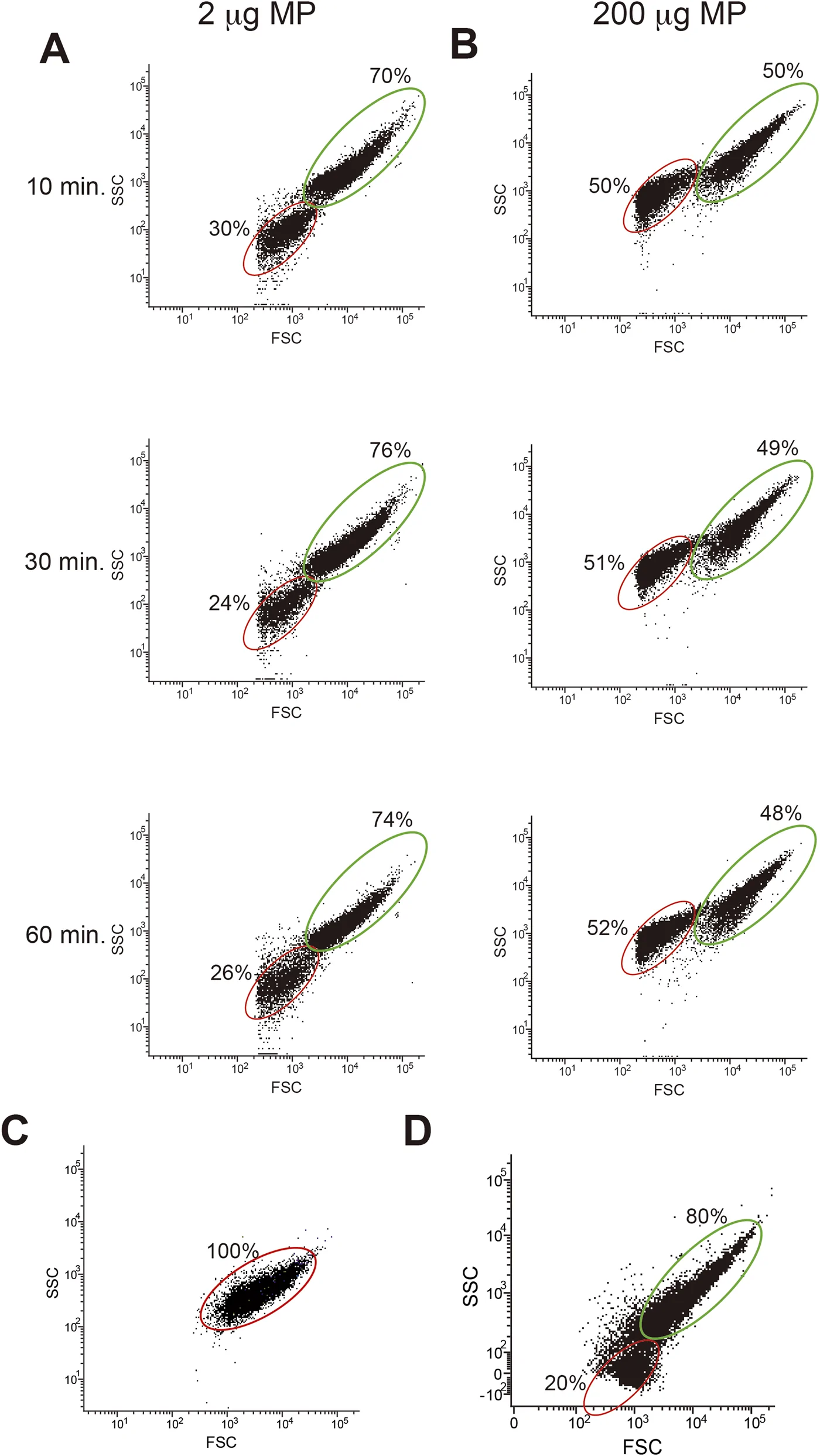
Effect of polystyrene nanoplastic (PS-NPs) concentration and thrombin on platelet morphology. **(A,B)** Flow cytometry analysis of washed human platelets exposed to varying concentrations of PS-NPs (2 μg in **(A)**, 200 μg in **(B)**) over a time course (10, 30, and 60 min). Dot plots show Forward Scatter (FSC; X-axis, reflecting cell size) versus Side Scatter (SSC; Y-axis, reflecting internal complexity). Two platelet populations are highlighted: resting (red gate) and activated-like (green gate), with the percentage of platelets in each population indicated. **(C)** Baseline scatter profile of unstimulated washed human platelets (0 min), showing the resting platelet population. **(D)** Positive control: Platelets stimulated with thrombin (10 U/mL) for 10 min. The thrombin-stimulated profile is used to delineate the activated-like population (green gate).

To determine whether platelet association with PS-NPs was accompanied by activation, FSC and SSC profiles were analyzed following nanoparticle exposure. Both low (2 μg) and high (200 μg) PS-NPs concentrations induced marked shifts in platelet scatter characteristics relative to untreated controls (Figure 2).

At 2 μg PS-NPs, 70%–76% of platelets transitioned into an activated FSC/SSC gate, characterized by increased SSC and reduced FSC, consistent with platelet shape change and cytoskeletal remodeling (Figure 2A). Exposure to 200 μg PS-NPs produced a broader and more heterogeneous scatter distribution, indicative of extensive structural remodeling and early microaggregate formation (Figure 2B).

The FSC/SSC activation profile induced by PS-NPs closely resembled that observed following thrombin stimulation (Figure 2D). Functional activation was further confirmed by concentration-dependent CD63 externalization, indicating dense granule secretion. Significant CD63 expression was detected at PS-NPs concentrations as low as 0.3 μg (Supplementary Figure S3).

### High-capacity association of PS-NPs with platelets

**FIGURE 3:**
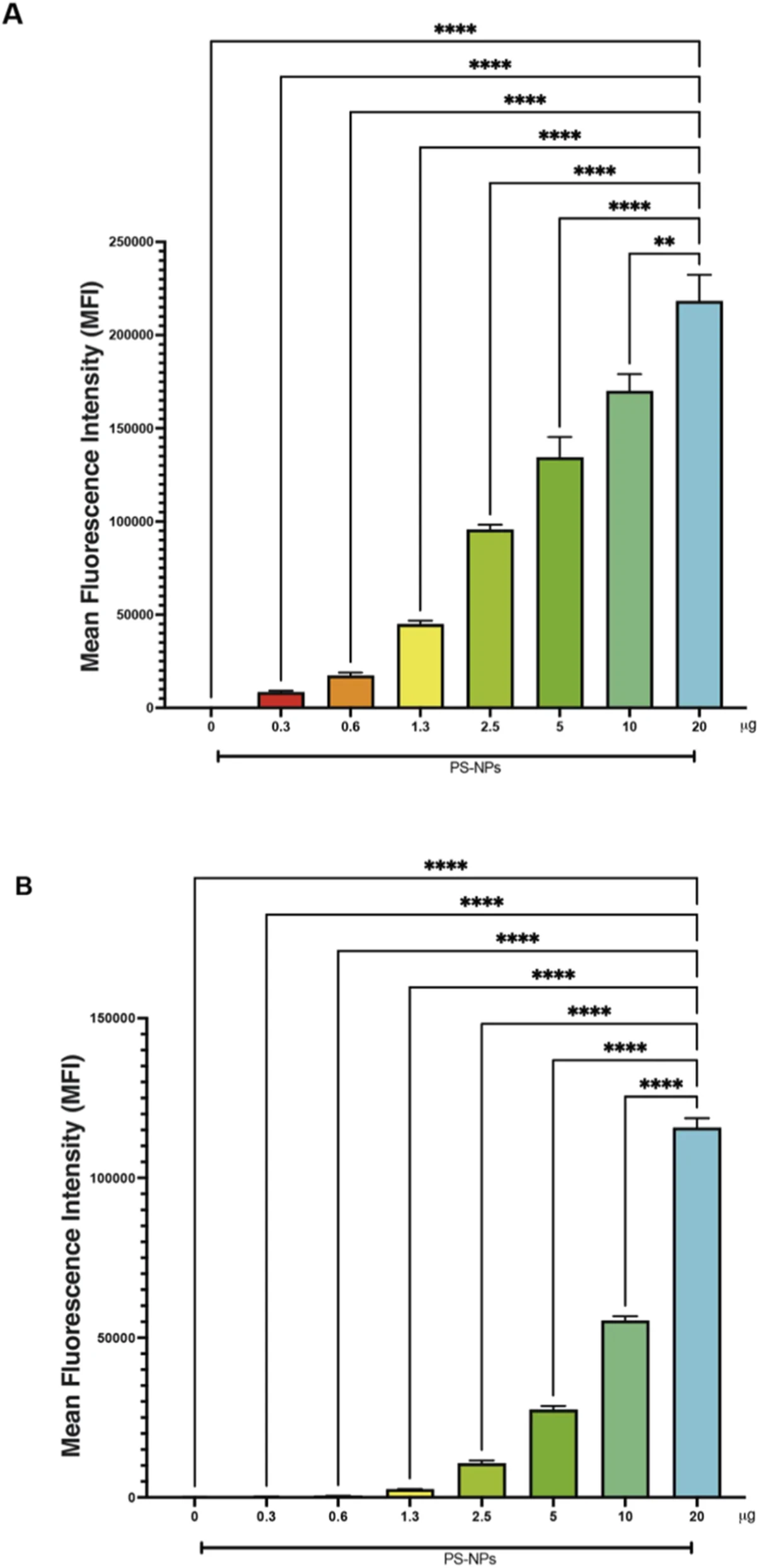
Dose-dependent polystyrene nanoplastic (PS-NPs) accumulation in washed human platelets. **(A)** Mean Fluorescence Intensity (MFI) of washed platelets exposed to varying concentrations of PS-NPs (0–20 μg) for a fixed 10-min period (optimized in Figure 1). **(B)** MFI of washed platelets exposed to 0–20 μg of PS-NPs for 10 min. MFI quantifies the average cellular load of associated PS-NPs. Data are presented as mean ± SEM. A clear dose-dependent relationship is observed. ****: p < 0.0001; **: p < 0.01 vs. 20 μg group by one-way ANOVA with Dunnett’s multiple comparisons test. Specific comparisons are indicated by brackets.

To assess the capacity of platelet–PS-NPs interactions, platelets were incubated with increasing PS-NPs concentrations (0.3–20 μg) for 10 min. Flow cytometric analysis revealed a dose-dependent increase in platelet-associated fluorescence across the entire concentration range (Figure 3).

Increasing PS-NPs concentrations were accompanied by progressive increases in SSC and fluorescence intensity (Supplementary Figures S4 and S5), consistent with enhanced platelet structural remodeling. These findings indicate that platelet association with PS-NPs occurs through high-capacity mechanisms that are not constrained by receptor-limited binding.

### PS-NPs facilitate calcium-dependent coagulation in whole blood

To examine the functional consequences of PS-NPs exposure under physiological conditions, whole blood coagulation assays were performed. Citrated whole blood incubated with 10 μg PS-NPs formed dense fibrin clots following calcium reconstitution.

**FIGURE 4:**
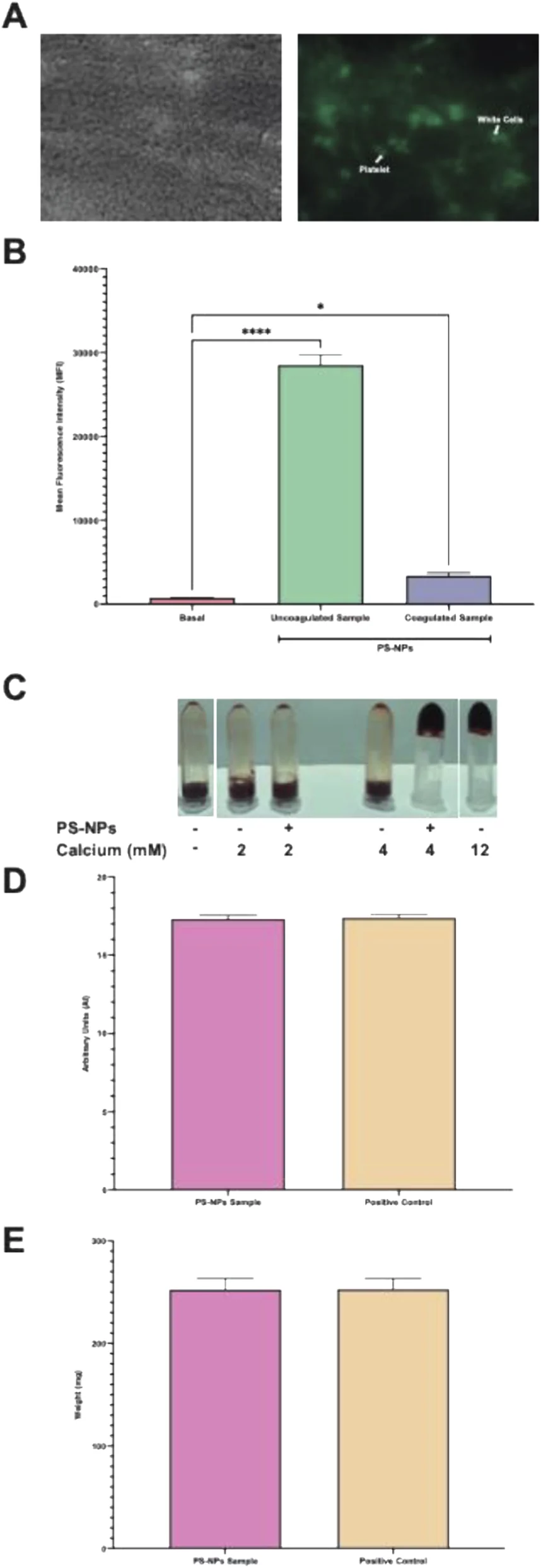
Polystyrene nanoplastic (PS-NPs) enhance coagulation and modulate fibrin clot formation. **(A)** Microscopic analysis of whole blood clots after 30 min of incubation with 10 μg of PS-NPs. Left: Bright-field image shows dense fibrin mesh trapping blood cells. Right: Fluorescence image confirms PS-NPs incorporation within the clot matrix. **(B)** MFI of PS-NPs in the liquid phase after 30 min of whole blood incubation. The MFI of the Basal (no PS-NPs) sample is compared to the supernatant of uncoagulated and coagulated blood samples. The reduction in MFI in the coagulated sample indicates PS-NPs sequestration within the fibrin clot. **(C)** Test tube assay demonstrating calcium (Ca^2+^)-dependent pro-coagulant activity of PS-NPs. Whole blood samples were incubated with or without 10 μgPS-NPs at various Ca^2+^ concentrations (0, 2, and 4 mM). Coagulation is assessed by the inability of the sample to flow when the tube is inverted. PS-NPs induce complete coagulation at 4 mM Ca^2+^. **(D,E)** Quantification of fibrin clot properties in the presence of microplastics. **(D)** Arbitrary size (AU) of fibrin clots. **(E)** Weight (mg) of fibrin clots. Data are shown as mean ± SEM. *: p < 0.05, ****: p < 0.0001 vs. Basal control by one-way ANOVA with Dunnett’s multiple comparisons test. Positive control (4 mM Ca^2^+).

Fluorescence microscopy demonstrated the incorporation of PS-NPs within the fibrin network (Figure 4A). Consistent with this observation, fluorescence measurements of the supernatant revealed a marked reduction in the PS-NPs signal following coagulation (Figure 4B), indicating sequestration of nanoparticles into the clot.

Macroscopic evaluation revealed that clot formation occurred exclusively when 4 mM Ca^2+^ was combined with PS-NPs, as evidenced by a stable clot that retained its position upon tube inversion (Figure 4C). In contrast, 4 mM Ca^2+^ alone or PS-NPs in the absence of calcium were insufficient to initiate coagulation. Importantly, this experimental profile remained unchanged over a 24-h observation period, during wich all other conditions failed to coagulate and remained liquid. The resulting clots induced by the nanoparticles were structurally robust and comparable to the positive control (12 mM Ca^2+^) (Figures 4C–E).

### Microscopic confirmation of rapid platelet–PS-NPs association

**FIGURE 5:**
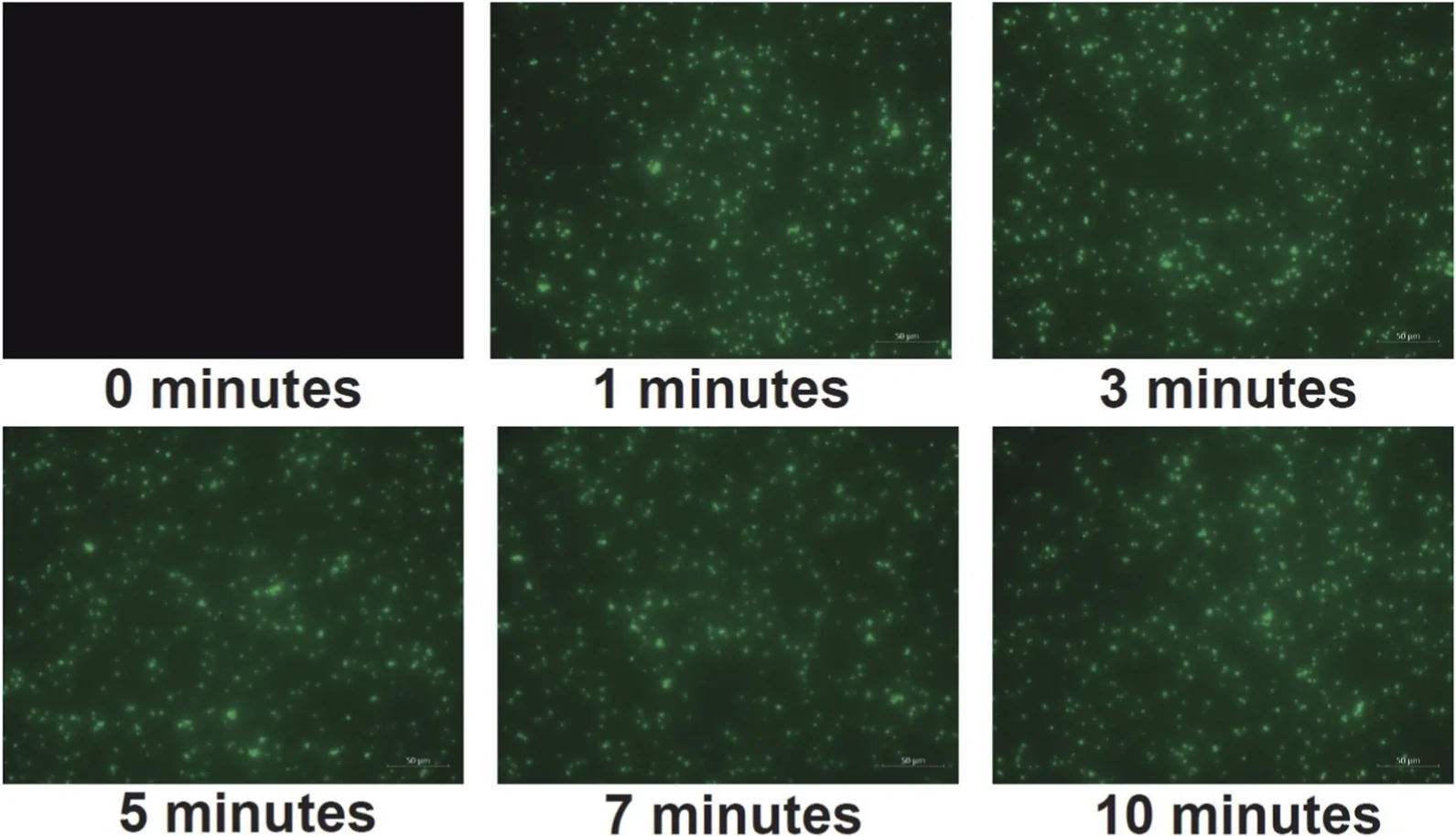
Time-dependent polystyrene nanoplastic (PS-NPs) interaction with washed platelets. Fluorescence microscopy images showing the kinetics of PS-NPs uptake by washed human platelets over 10 min (0, 1, 3, 5, 7, 10 min). The 0-min image is a negative control showing no background fluorescence. Distinct green fluorescent foci appear rapidly, confirming NP incorporation into platelets. Scale bar = 50 μm.

Fluorescence microscopy was used to independently validate the rapid kinetics observed by flow cytometry. Platelet-associated PS-NPs were detectable within 1 min of exposure and increased over time (Figure 5). The presence of platelet morphological changes and intracellular fluorescence provides preliminary evidence of rapid platelet-nanoplastic interactions.

## Discussion

In this study, we demonstrate that human platelets rapidly associate with PS-NPs, triggering activation, cytoskeletal remodeling, and calcium-dependent procoagulant responses. Platelet–PS-NP interactions occurred within minutes, were of high capacity, and induced functional activation comparable to that of thrombin. These findings identify platelets as direct cellular targets of circulating NPs and establish a mechanistic link between nanoscale particulates and thrombotic potential.

The rapid kinetics suggest that platelet–PS-NPs interactions are driven primarily by physicochemical adsorption rather than classical receptor-limited binding (Bai et al., 2021). In line with prior nanoparticle research, the formation of a protein corona in biological fluids may enhance these interactions, thereby supporting a high-capacity mechanism for platelet sequestration of NPs (Shiwakot et al., 2022; Burgos et al., 2025; White, 1972).

Importantly, platelet association with PS-NPs resulted in *bona fide* activation rather than passive uptake (Lee, 2024). PS-NPs induced characteristic changes in scatter profiles, pseudopod formation, and dense granule secretion, indicating cytoskeletal reorganization and degranulation (Rolli, 2023; White, 1972; Vucetic et al., 2018; Ruggeri, 2002). The close resemblance between PS-NPs- and thrombin-induced activation profiles (Assinger, 2014) suggests convergence on canonical platelet signaling pathways, including calcium mobilization and actin remodeling (Varela-Lopez et al., 2025). However, specific signaling intermediates remain to be defined.

Levels of NPs contamination range from 1.48 particles/g in seafood, 0.44 particles/g in sugar, 0.10 particles/g in honey, 0.11 particles/g in salt, 32.27 particles/L in alcohol, 94.37 particles/L in bottled water, and 4.23 particles/L in tap water (Yee et al., 2021; EPoCitF, 2016; Cox et al., 2019). Airborne exposure has also been documented at approximately 9.80 particles/m^3^ (Yee et al., 2021; EPoCitF, 2016; Cox et al., 2019). Although these values are reported as particle number rather than mass concentration—and direct conversion to μg/mL is limited by variability in particle size and polymer density—they support the concept of continuous, low-dose human exposure through ingestion and inhalation (Yee et al., 2021). Several studies have demonstrated that NP particles can cross epithelial barriers, enter systemic circulation, and distribute to peripheral tissues, making blood cells—including platelets—direct targets of exposure (Yee et al., 2021; Schneider et al., 2009; Tomazic-Jezic et al., 2001).

The concentration range tested in our study (0.3–20 μg PS-NPs) was selected to model escalating exposure conditions. We observed a progressive increase in platelet-associated fluorescence across the entire concentration range after 10 min of incubation. The parallel increases in side scatter signal further indicate concentration-dependent structural remodeling. The absence of a plateau suggests that platelet-PS-NP interactions are not constrained by classical receptor-limited binding kinetics but rather reflect high-capacity mechanisms, potentially driven by physicochemical adsorption, membrane insertion, or protein corona–mediated interactions. Importantly, this linear association implies that even modest increases in circulating NPs burden, such as those that may arise from cumulative environmental exposure, could proportionally enhance platelet interaction and functional perturbation, reinforcing the biological plausibility of thromboinflammatory consequences under chronic exposure conditions. The dose-dependent nature of platelet activation suggests that PS-NPs may function as amplifiers of platelet responses. Platelets integrate multiple weak stimuli to reach an activation threshold, and particulate surfaces are known to potentiate procoagulant phenotypes. NPs may therefore lower the threshold for platelet activation, particularly in primed or inflammatory vascular environments (Leslie et al., 2022; Guo et al., 2024; Nawab et al., 2024).

The mechanistic relevance of thrombin generation by quantifying prothrombin fragment F1+2 as an integrated marker of coagulation activation by MP was reported (Maitz et al., 2025). Because F1+2 is released during the conversion of prothrombin to thrombin, its increase directly reflects upstream activation of the coagulation cascade and positions thrombin formation—rather than clot formation alone-as a central event. Weathered aromatic MP significantly elevated F1+2 levels, supporting the concept that environmentally modified particles act as procoagulant surfaces. Our findings align with this mechanism. Whole blood experiments showed that PS-NPs participate directly in coagulation in a strictly Ca^2+^-dependent manner, indicating facilitation of coagulation complex assembly rather than primary initiation (Lindstrom et al., 2011; Holinstat et al., 2007). Moreover, incorporation of PS-NPs into fibrin networks supports a catalytic and structural role within thrombi, analogous to other procoagulant particulate surfaces (Huang et al., 2025). Our data reinforce that surface-driven thrombin generation is a key mechanism underlying microplastic-induced thromboinflammatory responses, rendering thrombin and thrombin receptor signaling mechanistically central.

*In vivo* studies showed that circulating NPs are captured by immune cells, leading to microvascular obstruction and thrombosis (Leslie et al., 2022). Our findings provide a complementary platelet-centered mechanism that may act synergistically with immune cell–mediated effects (Zheng et al., 2024; Smyth et al., 2015). Activated platelets may adhere to NPs-laden leukocytes or fibrin structures, amplifying thrombus growth and stability (Shiwakot et al., 2022; Burgos et al., 2025).

The current study has limitations. PS-NPs were used as a defined model system, whereas environmental NPs are heterogeneous in composition and surface chemistry (Rolli, 2023). Additionally, *ex vivo* approaches cannot fully recapitulate *in vivo* flow conditions. Future studies will be required to define polymer-specific effects, exposure thresholds, and *in vivo* relevance.

In summary, human platelets rapidly associate with PS-NPs through high-capacity mechanisms that induce activation, degranulation, and facilitate coagulation. These findings identify environmental toxic compounds as direct modulators of platelet function, revealing a previously unrecognized particulate pathway that enhances thrombotic responses.

## Data Availability

The original contributions presented in the study are included in the article/Supplementary Material, further inquiries can be directed to the corresponding authors.
