# Supplementary material for: Interaction of nanoplastics with platelets: activation and fibrin clot formation
Source: Front Pharmacol. 2026 Aug 24;17:1897581. doi: 10.3389/fphar.2026.1897581 (PMC13547509; doi:10.3389/fphar.2026.1897581)
Supplement: Supplementary file 1 [file DataSheet1.pdf]

Methods section

Flow cytometric analysis

The flow cytometric characterization of platelet activation states was conducted in alignment with the methodological framework outlined in the main text. The platelet suspensions[5] were acquired on a BD FACS Lyric flow cytometer (BD Biosciences), with acquisition thresholds set to collect a minimum of 10,000 cellular events per sample. Optical signals, including forward scatter (FSC) and side scatter (SSC), were detected in logarithmic mode to ensure resolution across heterogeneous cell sizes and granularities[11]. All aqueous buffers were subjected to dual sterile filtration through 0.22 μm membranes before use. Washed platelet preparations were immunophenotyped using a fluorochrome-conjugated anti-human CD41 monoclonal antibody (APC-Cy7-A conjugate; BD Biosciences, San Jose, CA, USA) or CD63conjugated with phycoerythrin (PE), which confirmed a purity exceeding 99% in the gated population. Quantitative measurements were derived from mean fluorescence intensity (MFI) values[11].

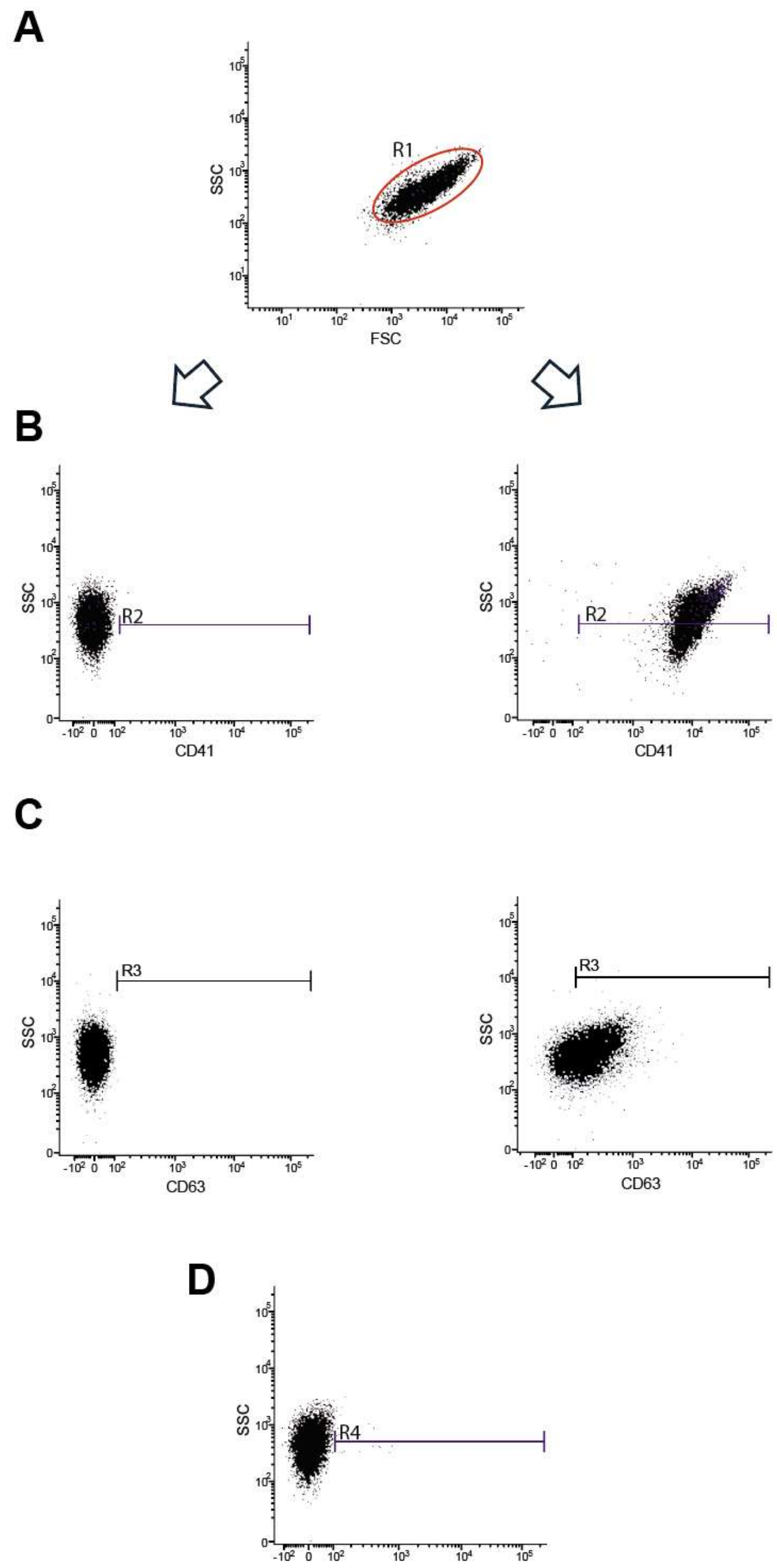

Supplemental Figure 1. High-Concentration Polystyrene Nanoplastic (PS-NPs) Association.

(A) Flow cytometry analysis of washed platelets exposed to 200 µg of fluorescently labeled PS-NPs over time (0, 10, 30, and 60 minutes). Dot plots show Side Scatter (SSC, Y-axis) versus PS-NPs fluorescence intensity (X-axis, logarithmic scale). Region R4 defines platelets positive for PS-NPs fluorescence.

(B) MFI of washed platelets following exposure to 200 µg of PS-NPs. Data are presented as mean ± SEM. ****: $p < 0.0001$.

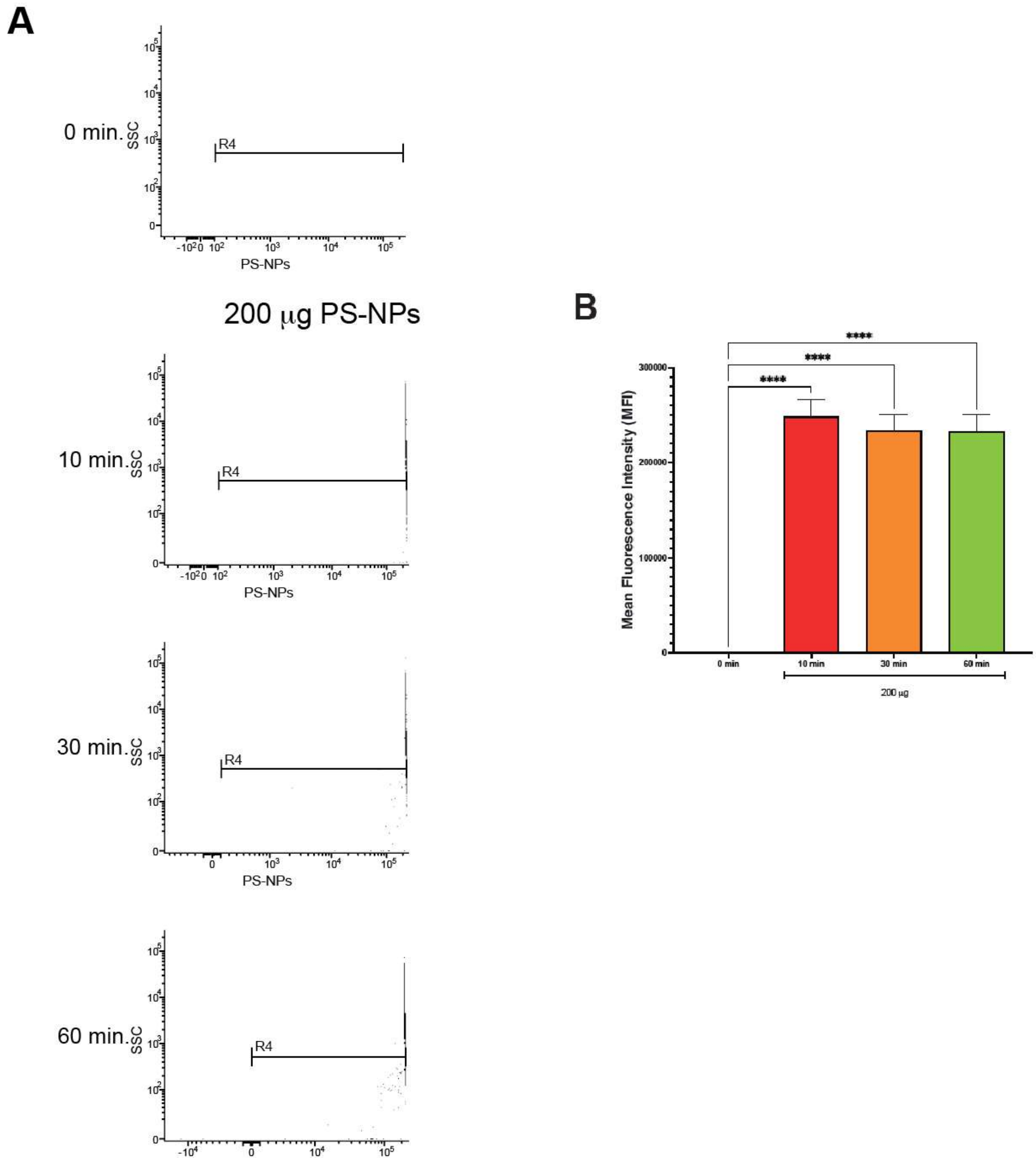

Supplemental Figure 2. High-Concentration Polystyrene Nanoplastic (PS-NPs) Association at 10-Minute Time Point.

(A) Flow cytometry analysis of washed platelets exposed to 200 µg of fluorescently labeled PS-NPs over time (0, 10, 30, and 60 minutes). Dot plots display SSC versus PS-NPs fluorescence intensity. Note the rapid shift of the platelet population towards the maximum detection range by the 10-minute time point. Region R4 defines platelets positive for PS-NPs fluorescence.

(B) MFI of washed platelets following exposure to 200 µg of PS-NPs. Data are presented as mean ± SEM. ****: $p < 0.0001$.

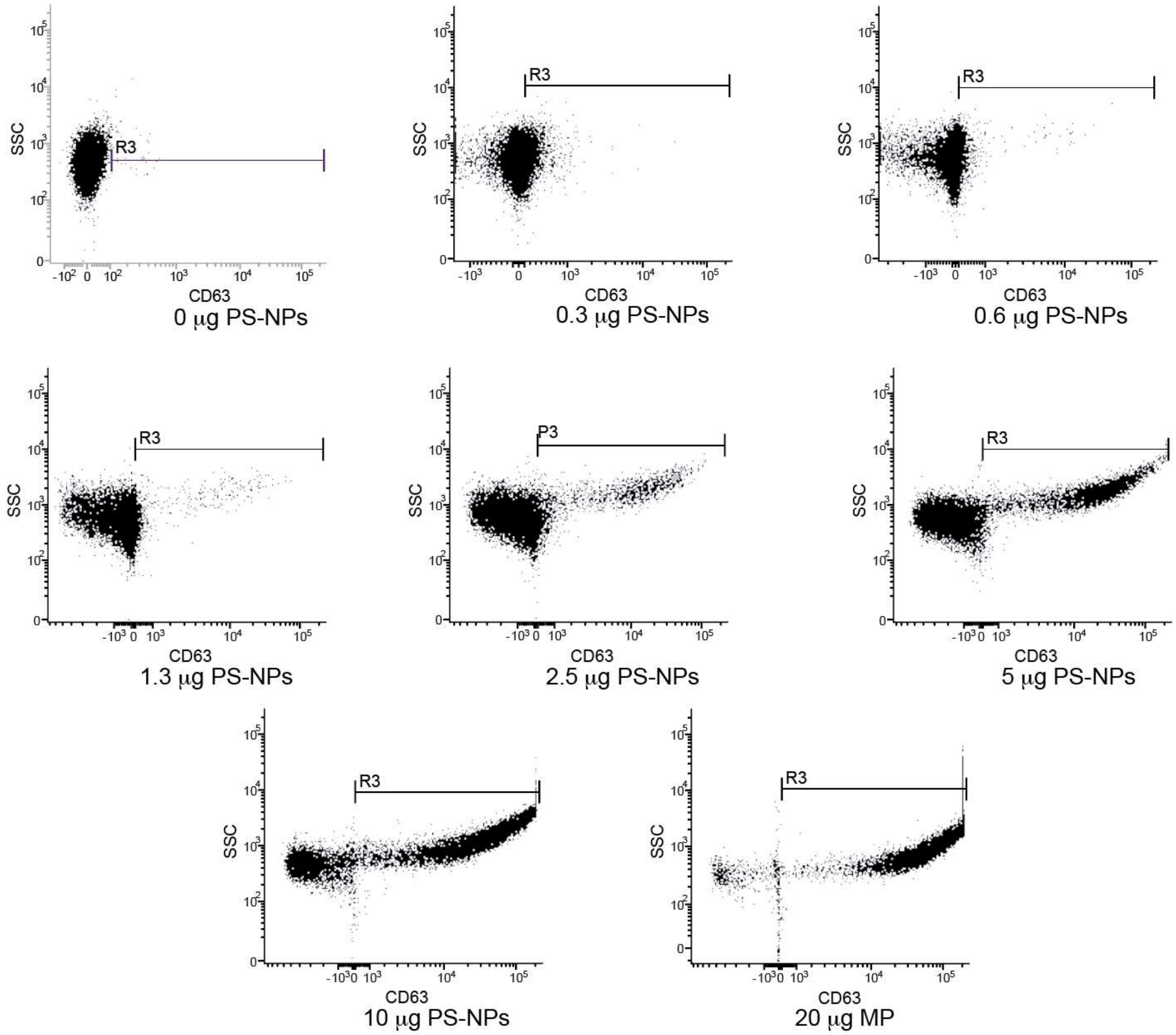

Supplementary Figure 3. Polystyrene Nanoplastic (PS-NPs) Dose-Dependent Platelet Activation.

(A) Representative flow cytometry scatter plots of washed human platelets incubated with increasing concentrations of PS-NPs (0 µg to 20 µg). Plots display SSC versus CD63 fluorescence intensity (X-axis, logarithmic scale), with CD63 serving as a marker for platelet activation. The R3 gate identifies platelets positive for CD63 expression. The shift towards higher CD63 fluorescence indicates a concentration-dependent increase in platelet activation, beginning at 0.3 µg.

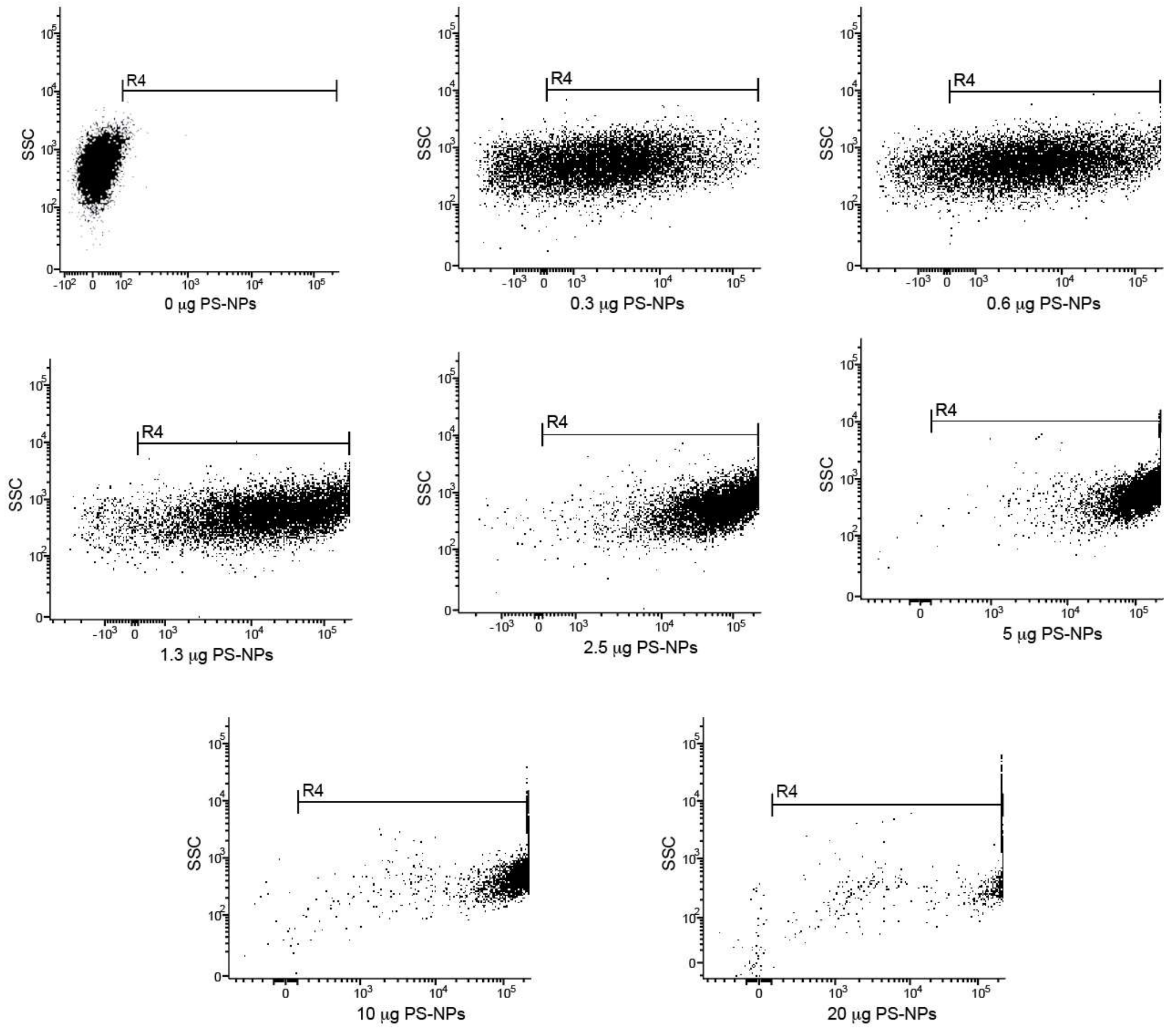

Supplemental Figure 4. Dose-Dependent Polystyrene Nanoplastic (PS-NPs) Uptake in Platelets.

Representative flow cytometry scatter plots of washed platelets showing dose-dependent increases in PS-NPs association. Plots display SSC versus PS-NPs fluorescence intensity (X-axis, logarithmic scale) for varying PS-NPs concentrations (as in Figure 3A). The shift toward higher fluorescence intensity indicates enhanced PS-NPs uptake with increasing concentration. The R4 gate defines the population of platelets positive for PS-NPs fluorescence.

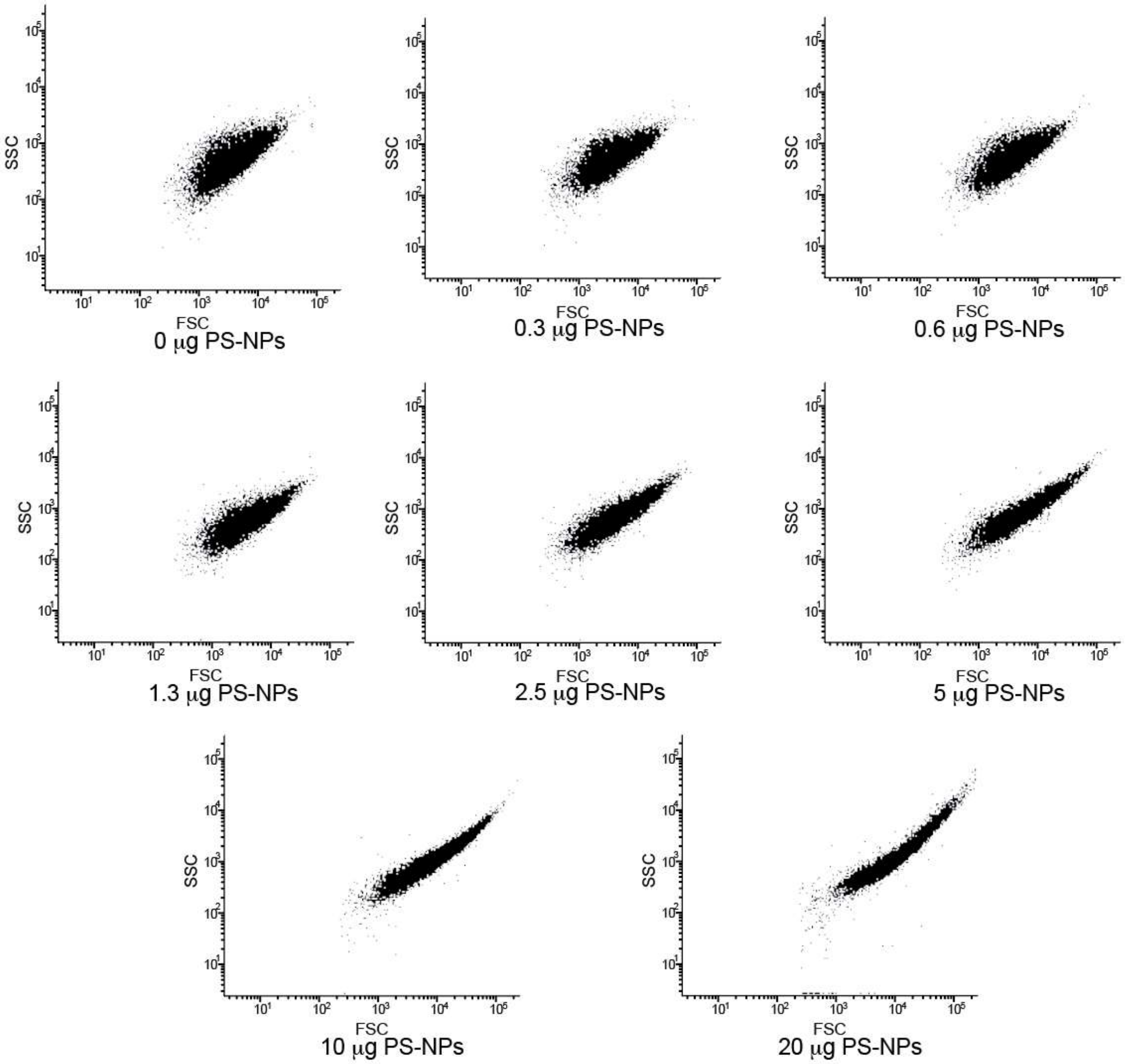

Supplemental Figure 5. Platelet Morphological Changes Induced by Increasing Polystyrene Nanoplastic (PS-NPs) Concentration.
Flow cytometry scatter plots showing Forward Scatter (FSC; X-axis, reflecting cell size) versus Side Scatter (SSC; Y-axis, reflecting internal complexity) of washed platelets exposed to increasing concentrations of PS-NPs (0 µg to 20 µg). A subtle shift towards higher FSC and SSC values with increasing PS-NPs concentration suggests a dose-dependent change in platelet morphology.
